# WNT Signaling in Neuroblastoma

**DOI:** 10.3390/cancers11071013

**Published:** 2019-07-19

**Authors:** Juergen Becker, Joerg Wilting

**Affiliations:** Department of Anatomy and Cell Biology, University Medical School Goettingen, Kreuzbergring 36, 37075 Goettingen, Germany

**Keywords:** sympathetic, development, cancer, embryonic, neural crest

## Abstract

The term WNT (wingless-type MMTV integration site family) signaling comprises a complex molecular pathway consisting of ligands, receptors, coreceptors, signal transducers and transcriptional modulators with crucial functions during embryonic development, including all aspects of proliferation, morphogenesis and differentiation. Its involvement in cancer biology is well documented. Even though WNT signaling has been divided into mainly three distinct branches in the past, increasing evidence shows that some molecular hubs can act in various branches by exchanging interaction partners. Here we discuss developmental and clinical aspects of WNT signaling in neuroblastoma (NB), an embryonic tumor with an extremely broad clinical spectrum, ranging from spontaneous differentiation to fatal outcome. We discuss implications of WNT molecules in NB onset, progression, and relapse due to chemoresistance. In the light of the still too high number of NB deaths, new pathways must be considered.

## 1. WNT Signaling

The name WNT is an acronym combining the *Int1* gene (see list of abbreviations at the end of the text) found in mice and the *wingless* gene from drosophila [1,2]. In mammals, we know of 19 WNT ligands today. They are palymitoylated proteins, which are secreted and can act in a paracrine or autocrine manner by binding to a multitude of receptors and co-receptors (reviewed in [3]). This offers a delicate spatial and temporal control for a variety of functions including regulation of cell proliferation and migration, the determination of cell polarity, regulation of cell survival and differentiation. Therefore, WNTs are important regulators of cell fate during embryonic development [4,5]. The multidimensional nature of this system ensures a comprehensive control of developmental processes. This is achieved not only by the spatial control of WNT ligand expression, but also by the versatile combination of receptors and co-receptors expressed on physiological targets and aberrant target cells. The network of interactions is not fully understood today, however, a great amount of data already exists [6]. WNT pathways are usually segregated into three branches. The first and most intensely studied pathway involves the ligands WNT1, WNT2, WNT3 and WNT8 and is often referred to as ’canonical’. These ligands bind to the LRP5/6 co-receptors together with receptors of the FZD family, which consists of 10 members. Upon binding to FZD/LRP receptor complexes, components of the steady degradation complex for β-catenin—including DVL, AXIN, GSK-3β and others—are recruited to the receptors, β-catenin degradation is blocked, causing its accumulation and translocation to the nucleus where it activates TCF/LEF-dependent transcription of target genes [7]. The receptors LGR4/5/6 and their ligands, the R-spondins, further enhance TCF/LEF-mediated WNT/β-catenin signaling [3,8,9]. This pathway is regarded to promote proliferation of cells by activation (or acceleration) of the cell cycle.

The other two pathways, the so-called planar-cell-polarity-pathway (PCP) and the Ca^2+^-regulated ’WNT/Ca^2+^ pathway’ are both independent of β-catenin and TCF/LEF signaling. They are activated by WNT4, WNT5 and WNT11, and involve FZD receptors as well as co-receptors such as ROR1, ROR2, and RYK (reviewed by [10]). The WNT/Ca^2+^ pathway is characterized by Ca^2+^ release upon activation, and involves the small GTPases RHOA and RAC. Then, it activates kinases such as PKC, JNK or CaMKII, which induce transcription factors like AP1 and NFAT [11,12]. The main effects are cytoskeletal rearrangement and migration (reviewed in [13,14]).

The PCP pathway is activated by the receptor tyrosine kinases ROR1, ROR2 and RYK, which may act alone, or as co-receptors in conjunction with FZDs (reviewed by [10]). It acts by regulating the degradation rates of the transmembrane receptors VANGL1/2, which interact with CLSR2, the human homologue of *Drosophila* Fmi (Flamingo), to orientate epithelial as well as mesenchymal cells during the development and regeneration by cell-cell interactions according to a WNT gradient [15]. This also affects directed migration, including convergence-extension movements, in response to WNT gradients. RYK seems to have a special function in WNT signaling. Its extracellular domain is highly similar to the WNT-inhibitory-factor WIF, suggesting that it may have a role (i) as a receptor (ii), as a decoy receptor or (iii) as a co-receptor that binds ligands and presents them to signaling receptors (reviewed in [10]). The receptor tyrosine kinase PTK7 is a regulator of PCP signaling that interacts with ligands, co-receptors and intracellular signaling molecules, providing crucial fine tuning of cell behavior (review in [16]).

The separation of WNT signaling into distinct pathways may be feasible to generate research approaches and to concentrate on functional aspects in a given cell-type or organism. However, there is emerging evidence that WNT signaling is much more sophisticated than initially expected (reviewed by [3,10]). Several WNT signaling molecules are involved in more than just one of the three pathways, and some of the ligands and receptors seem to be redundant as they may substitute for each other. For example, ROR1/2 are (co-)receptors in WNT5A signaling, and only knock-out of both of them approximately mimic the complete phenotype of the Wnt5a knock-out in mice (details below). The combination of receptor and co-receptor on a given cell seems to determine which WNT pathway is activated; for example when WNT3A and WNT5A compete for FZD receptors, the availability of the co-receptors LRP5/6 or ROR1/2 is decisive for which pathway becomes activated [17,18]. The first evidence for this was provided about 20 years ago by showing that WNT5A—a typical non-canonical ligand—can activate the β-catenin-dependent pathway when FZD4 or FZD5 and LRP5 are over-expressed [19]. Similarly, but in the opposite direction, GSK-3β—a central kinase of the WNT/β-catenin pathway—also provides signaling cues independent of β-catenin upon binding of WNTs to the FZD/LRP6 receptor complex [20]. Consistently, Lrp6 was shown to act together with non-canonical Wnt5a during neural tube closure in murine embryos [21].

In sum, canonical and non-canonical WNT signaling pathways have their specific roles in the regulation of cell behavior, but they also interact, mostly in a mutually inhibitory fashion [22]. Thereby, canonical WNTs usually control cellular proliferation, whereas the non-canonical WNTs affect cell polarity and motility. However, exceptions to these rules have regularly been shown.

## 2. Neuroblastoma

Neuroblastoma (NB) is a pediatric tumor of the developing sympathoadrenal system. Severe, unfavorable NB acquire activating mutations in the *ALK* gene or gene amplifications of *MYCN* [23,24]. However, NB in patients less than about 18 months of age often regress spontaneously or under mild interventionif they do not bear specific risk factors [25,26]. Only recently, PCF11 has been discovered to influence alternative polyadenylation [27]. Postnatal down-regulation of PCF11 induces differentiation in sympathoadrenal neuroblasts, and low expression correlates with spontaneous regression and favorable prognosis.

Early section studies showed that NB often exist prenatally with an incidence much higher than the clinically diagnosed cases [28,29]. This gave rise to the hypothesis that NB may regress spontaneously in many cases and remain clinically silent. This is supported by studies in the 80s and 90s conducted in Japan, Europe and North America with the objective of an early detection of NB patients by the screening of catecholamines in urine during the first months of life [30,31,32]. Even though the screening increased the number of detected NB dramatically, the number of fatal outcomes could not be reduced, and in the end the trial was stopped to avoid adverse effects of clinical over-diagnostics [33]. Of note, spontaneous differentiation and regression of the lesion is a feature usually restricted to low grade L1+2 (stage 1+2) and stage MS (4S) tumors in very young patients [34,35,36]. About 98% of NB occur in children younger than 10 years. Thereby, prognosis worsens and spontaneous differentiation becomes rare with increasing age [25,37,38]. Accordingly, there are two strategies in NB treatment that reflect this phenomenon: one is the wait and see approach, meaning that low grade NB that demand no urgent therapy are closely monitored, hoping for spontaneous regression; the second is the use of differentiating drugs such as retinoic acid derivatives to induce tumor cell differentiation in low grade tumors, usually after surgical intervention and in combination with chemo- or radiotherapy when necessary [25,39,40,41].

## 3. The Origin of Neuroblastoma

To understand the differentiation ability of NB, its origin must be considered. NB is widely accepted as an embryonic developmental malignancy derived from progenitor cells originating from the sympathetic neural crest (reviewed by [42]). These cells are (pre-)determined to form sympathetic ganglia, the adrenal medulla and paraganglia, which serve as oxygen-sensors capable of releasing adrenalin into the fetal circulation to increase cardiac activity under hypoxic conditions (for review see [22,43]). These paraganglia regress after birth when hypoxia can be balanced by increased respiration. It has been reported in mice that neural crest stem cells (NCSC) extensively migrate through the body and can be found in bone marrow, liver, mesonephros, blood-stream and other organs [44]. Even though it has remained unclear which of the NCSC are sympathetic progenitor cells, virtually all emerging NC cells were marked in this study regardless of their localization in the NC (even cranial and cardiac NC were marked; compare [22]); therefore, this study provides a hint to the etiology of NB. This applies especially to the etiology of the (former) 4s stage (now known as MS), which is found in patients under 1 year of age, where metastases are restricted to dermis, liver and bone marrow (reviewed in [26,45]). It has been assumed that dermal ’metastases’ are a multifocal disease rather than lesions emerging from a primary tumor. In other words, the cells might be siblings rather than offspring [46].

Both growth and spontaneous regression of NB seem to recapitulate the developmental program of sympathoadrenal progenitor cells. Considering that many NB do not attract clinical attention and many of those that require clinical intervention regress spontaneously or respond well to mild therapy, the drivers of malignant NB, such as ALK and MYCN, seem to be acquired after the differentiation program failed. Of note, tyrosine hydroxylase promoter-targeted Mycn over-expression has been shown to induce NB in mice, indicating that it is a sufficient and independent factor that defines a highly malignant subclass of tumors [47]. In nude mice, lentiviral transduction of sympathoadrenal progenitor cells for Mycn over-expression revealed an increase in proliferation, but did not induce tumor forming capability [48]. Therefore, MYCN expression seems to increase malignancy of cells that have acquired transforming changes, but on its own it is not sufficient to induce malignant transformation of healthy progenitor cells. It seems to be worth having a closer look at molecules that orchestrate the development of the sympathetic nervous system, adrenal medulla and paraganglia, and may be causal for non-MYCN or non-ALK-driven NB or may initiate the malignant progression. In this study, we focus on the WNT pathway.

## 4. Wnt Involvement in Sympathetic System Development

NB evolve from progenitors of postganglionic sympathetic neurons. These emigrate from the sympathetic neural crest (NC), which spans a large portion of the trunk NC. It overlaps with the adrenal NC, but is distinctly separated from the cranial, vagal and sacral NC. As the developmental aspects were extensively reviewed recently [22], only a short overview is given here. Of note, also chromaffin cells of the adrenal medulla, sympathetic and adrenal glia, neurons and glia of the dorsal root ganglia and melanocytes originate from this part of the NC [43,49].

**Figure 1:**
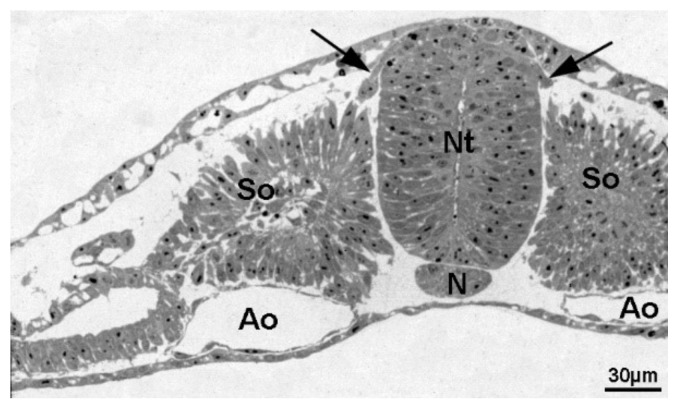
Transverse section of a chick day-2 embryo showing migration of neural crest cells (arrows) from the neural tube (Nt). Ao: Aorta; N: Notochord: So: Somites.

The sympathoadrenal progenitors migrate ventrally via two routes (Figure 1; for review see [22]). The first route is straight ventrally between the neural tube and the somites. The cells stop at the ventral side of the dorsal aorta and differentiate into pre-vertebral sympathetic ganglia. The second route guides the cells through the cranial half of each somitic sclerotome. Some progenitors stop here to form the dorsal root ganglia, whereas the sympathetic precursors are guided through the sclerotome by repulsive EphrinB1/EphB2 and Sema-3F/Nrp2 signals. They migrate to the dorso-lateral aspect of the dorsal aorta where they form ganglia of the paravertebral sympathetic trunk. At this stage of development, WNT signaling has not been reported to play a role for sympathetic precursor migration and determination. In contrast, sensory neuronal precursor cells that stop within the somites express WNT and neurotrophin receptors. They may actually be pre-determined by neurotrophin3 and WNT ligands from the neural tube to form sensory ganglia. For ganglia formation proper, Delta/Notch signaling seems to be necessary. Sympathetic cells that do not participate in this process—but migrate through the somite—do not express WNT or neurotrophin receptors [50,51]. Therefore, at this developmental stage the absence of WNT signaling seems to be obligate for the generation of sympathoadrenal cells. It may therefore be allowed to speculate that NB clinically presenting with compression of the spinal cord (hourglass syndrome) may have evolved due to mis-expression of WNT or neurotrophin receptors, thereby stopping their migratory activity together with the precursors of sensory neurons. To the best of our knowledge, no data exist on this topic. However, cells that differentiate in the wrong environment may be susceptible to malignant transformation [52]. A wrong environment may provide cues that lead to misbehavior of cells (for review see [53,54]). Such signals include WNT ligands and WNT inhibitors such as DKK1, as well as cytokines and chemokines, which in the adult are often provided by leukocytes but have additional functions for embryonic mesenchymal cell migration [55,56]. The final guidance and differentiation of sympathetic progenitors of the sympathetic ganglia and adrenal medulla is under control of BMP secreted from the dorsal aorta and the adrenal cortex [43,57,58].

When sympathetic neurons have entered the post-mitotic phase, axons start growing toward their target organs. HGF, Artemin, and NGF are involved in this process. NGF released from target organs induces transcription of key sympathoadrenal molecules like tyrosine hydroxylase (TH), dopamine beta-hydroxylase (DBH), and also WNT5A [59]. Autocrine WNT5A subsequently induces axon branching to multiply neuronal effects on target cells. Defects in NGF/TRKA or WNT5A/ROR1/2 signaling interferes with the innervation of target organs, and failure of innervation leads to perishing of neurons [60,61,62]. Recently, Li et al. found mutations of axon guidance molecules in a cohort of 64 primary NB, indicating that this type of cellular pathfinding is an important aspect of sympathoadrenal development and disease [63]. It has to be pointed out here that the expression of TRKA in NB is a favorable prognostic marker, whereas TRKB expression (ligand: BDNF), which is by today’s knowledge not involved in sympathetic differentiation, is unfavorable [62,64]. It may be speculated that TRKB expression is a cause of mal-differentiation that leads to cancer.

## 5. WNTs and Neuroblastoma

In the past, WNT signaling in NB did not gain much attention, although the first studies appeared in the mid-2000s. Then, it took almost 20 more years to increase evidence and interest for WNT signaling in NB. As noted above, β-catenin-dependent (canonical) and β-catenin independent (non-canonical) pathways must be considered.

### 5.1. WNT-β-Catenin Signaling in NB

The WNT/β-catenin signaling has shown to influence NB. An early microarray study, however, did not report differentially expressed genes obviously involved in WNT signaling [65]. Nevertheless, implications of WNT/β-catenin have been described. Not surprisingly, WNT/β-catenin signaling components were shown to be involved in NB proliferation. Especially in *MYCN* non-amplified cell lines, enhanced WNT/β-catenin signaling was shown to increase MYCN levels [66]. Thereby, the ’canonical’ ligands WNT1, WNT6, WNT7A and WNT10B are upregulated, accompanied by higher expression of FZD2 and LRP5, supporting an important role for them in NB. In the SH-SY5Y NB cell line, silencing of WNT1 expression by RNAi significantly reduces cell viability [67]. Knock-down of FZD2 was recently shown to inhibit proliferation of the NB cell lines SK-N-AS and SK-N-DZ, as well as WNT3A or WNT5A-mediated migration in SK-N-DZ and WNT5A-mediated migration in SK-N-AS [68]. In both cases the activation of RAC1—a component of the PCP signaling—was involved, highlighting the difficulties of thinking in plain canonical/non-canonical categories. Repeated injection of FZD2 siRNA into NB xenografts significantly inhibited tumor growth in mice and led to decreased levels of β-catenin, MYCN and cyclin D1 protein. Similarly, the silencing of MYCN expression via RNAi in *MYCN*-amplified NB cell-lines reduced viability due to increased apoptosis and increased expression of DKK1, thus inhibiting WNT/β-catenin signaling [69].

Very recently, TRIM 59, a transmembrane protein of the endoplasmic reticulum, has been shown to regulate proliferation and β-catenin levels in NB cells [70]. Knock-down of TRIM59 inhibits proliferation and induces apoptosis in SK-N-SH and SH-SY5Y NB cells. This effect is partially rescued by LiCl, an inhibitor of GSK-3β and thereby inducer of WNT/β-catenin signaling. Reversely, the enhancement of cell proliferation by TRIM59 over-expression could be attenuated by application of XAV935, a tankyrase1/2 (TNKS1/2) inhibitor that blocks WNT-β-catenin-mediated transcription, but does not alter CRE, NF-κB or TGF-β signaling. TRIM59 over-expression also up-regulated Survivin, c-MYC and β-catenin and down-regulated apoptosis pathway proteins such as BIM and BAX. Again, application of XAV935 reversed these expression patterns. Earlier, induction of apoptosis in NB by XAV939, another tankyrase1 inhibitor, has also been reported [71].

LGR5 has been identified as a marker of aggressive NB and was shown to be elevated in relapsed NB [72]. It is regarded as a stem cell marker, and pronounced LGR5 expression is found in aggressive sub-types of many cancers. In NB, LGR5 expression correlates with aggressiveness, is associated with MYCN amplification, and also with NB progression and recurrence (reviewed by: [73]). Binding of R-spondins to LGR transmembrane receptor has been shown to enhance WNT/β-catenin signaling [8]. Knock-down of LGR5 in SK-N-BE(2), SK-N-AS and SH-SY5Y NB cells increases apoptotic cell death independently of β-catenin [74]. Application of WNT3A, together with R-spondins in TOPFLASH reporter assays, resulted in increased pathway activation. Moreover, in LGR5 knock-down, phosphorylation of MEK/ERK MAP-kinase signaling was reduced and also the AKT/mTOR pathway was affected negatively. RAS/RAF mutations that act upstream of MAP-kinase signaling have recently been identified to be frequently acquired during relapse in NB and promote an unfavorable phenotype [75]. WNT3A/RSPO2 signaling has been shown to induce changes in the transcriptome of NB cell lines—not only affecting WNT-signal molecules but also BMP4, Cyclin D1 and phosphorylation of the RB protein—indicating a crucial role for differentiation or progression of NB [76].

### 5.2. WNT Signaling and Chemoresistance

WNT/β-catenin signaling is also involved in chemoresistance of NB. Comparison of doxorubicin-resistant and non-resistant LAN1 and IGRN1 NB cells revealed higher expression of MDR, FZD1 and other components of WNT/β-catenin signaling in the resistant subclones. Elimination of FZD1 by shRNA in these cells reduced MDR1 expression and restored sensitivity to doxorubicin [77].

In SK-N-SH a subset of CD133^+^ cells could be identified, and they showed increased resistance to doxorubicin compared to CD133^-^ cells [78]. Notably CD133 is widely accepted as a marker for tumor stem cells in many cancers (reviewed in: [79]). The same group later compared CD133^+^ with CD133^-^ cells, each treated with doxorubicin, and detected differential expression of WNT signaling molecules. Especially APC, DVL2, FZD4, FZD6, CSK1 and LEF1 were associated with CD133^+^ cells under doxorubicin treatment. These cells were shown to exhibit lower sensitivity to doxorubicin, but could be sensitized by concomitant application of XAV 939, which inhibits WNT/β-catenin-mediated transcription [78]. Additionally, doxorubicin treatment of CD133^+^ cells increased β-catenin and pGSK-3β levels as compared to CD133^-^. Similar results were shown for SH-SY5Y and IMR32 cells treated with XAV939. Treatment led to decreased β-catenin abundance and enhanced sensitivity to doxorubicin [80].

**Figure 2:**
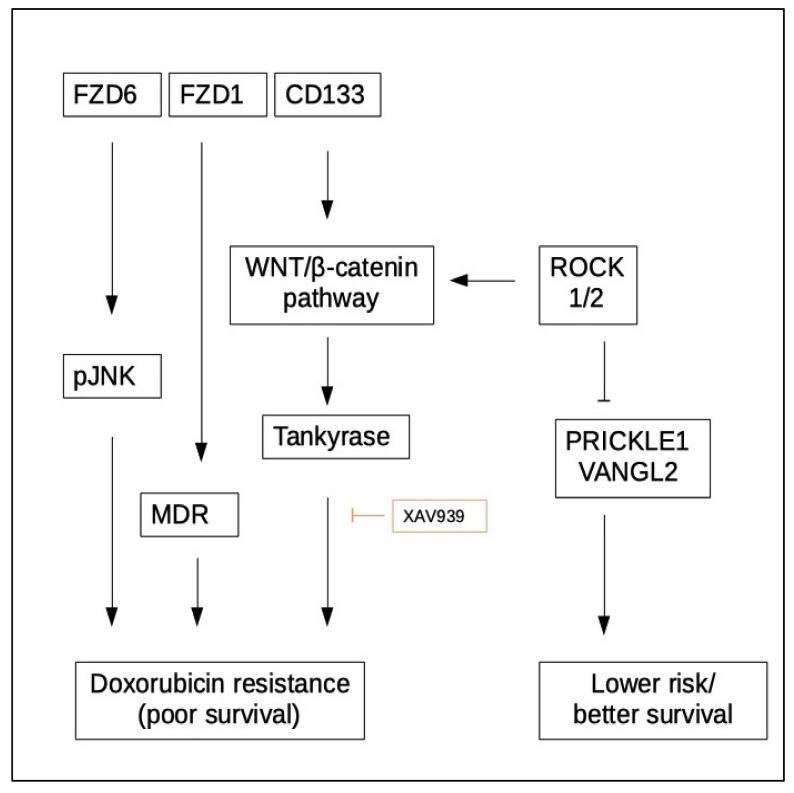
WNT pathway molecules and their involvement in chemoresistance in NB. For detailed, information please see text.

Together these results reveal that canonical WNT signaling is a major component for chemoresistance in NB, and is associated with tumor cell stemness and expression of stem cell markers. However, all studies are focused on doxorubicin and it cannot be excluded that resistance to other chemotherapeutics differs (Figure 2).

### 5.3. GSK-3β, Central, not Only in WNT Signaling

**Figure 3:**
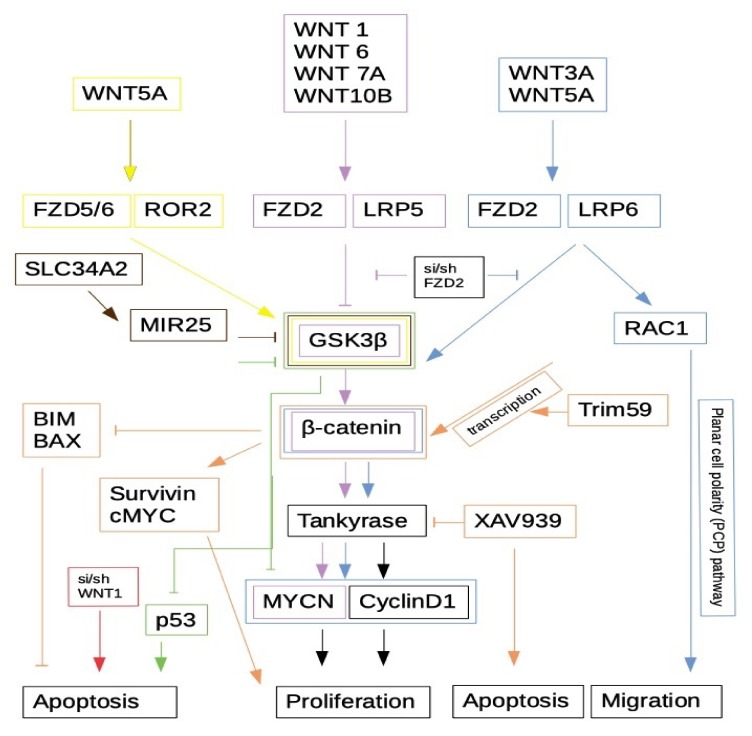
Synthesis of β-catenin involvement in NB as provided by several authors. Blue: [68]; purple: [66]; yellow: [17,18,91,92]; brown: [93]; green: [88]; orange: [70]; red: [67]. For detailed information please see text.

One of the first studies on this topic was published by Orme et al. 2003, who used the N2A mouse NB cell line and showed that GSK-3 is involved in ’neurite’ outgrowth, a widely accepted sign for NB differentiation [81]. They also indicated that signaling is independent of β-catenin and proposed an independent pathway, even though GSK-3β is widely regarded a key enzyme for β-catenin stability and canonical WNT signaling (Figure 3). The involvement of GSK-3β in neurite outgrowth was confirmed by Zhi et al., who could reverse outgrowth with the (specific) inhibitor BIO [82]. However, in contrast to Orme et al., they could show that β-catenin is involved by introducing a constitutively activated (phosphorylation site-mutated) β-catenin variant in N2A cells, no longer making them susceptible for GSK-3β inhibition. The obstacle with GSK-3 is that there are two isoforms—alpha and beta—which are transcribed from closely related but independent genes and cannot replace each other, as shown in knock-out experiments [83]. Furthermore, GSK-3β exists in two isoforms (GSK-3β1 and β2), but only isoform β1 seems to have significant affinity to Axin, whereas the brain-specific isoform β2 seems not to be involved in WNT signaling [84]. Therefore, GSK-3 is not only involved in WNT signaling, but is a component of plenty of other pathways (for review see: [85,86]. The inhibitors usually studied seem to affect both isoforms, and it is not overall clear yet which, isoform phosphorylates targets which (reviewed by: [87]). This illustrates why GSK-3 inhibitors are poor drugs and cause extensive side effects. GSK-3 inhibition is nevertheless an interesting strategy as it was shown to down-regulate MYCN protein levels by decreasing mRNA stability and inducing apoptosis—at least partly via p53 [88]. In xenografts of the murine neuroblastoma cell line N2A inhibition of GSK-3β reduces tumor growth by induction of G2/M cell cycle arrest [89]. Of note, it seems that PI3K signaling via AKT and GSK-3 does not affect WNT signaling as GSK-3 seems to be protected from this pathway by its complexing with Axin [90].

Recently SLC34A2, which is associated with lung and colorectal cancer malignancy, was show to be up-regulated in spheroids of SH-SY5Y cells and to produce direct effects by inducing MIR-25, which leads to the degradation of GSK-3β and accumulation of β-catenin, thus increasing proliferation and expression of stemness markers [93]. Knock-down of miR-25 or over-expression of GSK-3β inhibited these SLC34A2 effects. GSK-3 is a multifunctional enzyme connecting numerous pathways, and, as shown in the following paragraph, is involved in crosstalk between canonical and non-canonical WNT signaling.

### 5.4. Non-canonical WNT Signaling in NB

Non-canonical WNT signaling has shown to be involved in both progression and suppression of several human cancers (reviewed in: [94]). Decreased expression of WNT5A has been observed in cells from metastatic sites as compared to the xenografted primary tumor. In patients, samples of favorable tumors presented a statistical increase in WNT5A expression. However, only a few individual samples showed this elevation, and the larger number of lesions were similar to the unfavorable group [95]. WNT5A and WNT11 are the typical ligands that induce PCP-signaling. Only recently, high expression of the PCP pathway molecules PRICKLE1 and VANGLE2 has been found to correlate with low-risk NB [96]. In a wide panel of cell lines, knock-down of PRICKLE1 and VANGLE2 accelerated proliferation of NB cell lines, while over-expression reduced proliferation. Inhibition of ROCK1/2, which are down-stream kinases of RhoA in the PCP pathway, induces up-regulation of PRICKLE1 and inhibits β-catenin-dependent WNT signaling. Of note, the study reveals that the heterogeneity of NB and the complexity of the WNT pathways lead to different results in various cell lines. However, in patients, both high PRICKLE1 and VANGLE2 correlate independently, with better survival and non-high-risk NB.

PCP signaling is initiated by binding of WNT5A or WNT11 to ROR1, ROR2 or RYK receptor tyrosine kinases. Wnt5a knock-out mice show severe craniofacial defects and malformation of dermal lymphatics [97] (reviewed in [10]). A milder phenotype is caused by the loss of Ror2, whereas Ror1/2 double knock-out mice recapitulate the phenotype of Wnt5a-deficient mice. Interestingly, ROR2 and FZD5/6/7 form a complex with WNT5A and recruit components of the canonical signaling such as DVL, Axin and GSK-3β, suggesting competition with this pathway [17,18,95,96]. Axin and GSK-3β have been shown to activate neurite outgrowth in NB cells via β-catenin independent signaling [81]. RYK, the third receptor of the family, may have a regulatory function in WNT5A signaling, since Ryk/Vangle2 double knock-out mice largely recapitulate the Wnt5a-null phenotype [15].

FZD6 expression correlates with poor survival in NB patients and is a marker of NB stem cells [98]. High expression in mouse NB cells correlates with doxorubicin resistance, enhanced sphere formation and increased tumor growth in nude mice. JNK phosphorylation is enhanced in FZD6-positive cells, suggesting action via PCP-signaling.

FZD2 is a known receptor in WNT5A-mediated signaling and has been shown to correlate with metastases and poor prognosis in breast, colon and squamous cell carcinoma [99,100,101]. In NB, FZD2 is important for the proliferation of SK-N-AS and SK-N-DZ cells. The knock-down with siRNA decreases proliferation in both cell lines [68]. Interestingly, FZD2 knock-down also abrogates WNT3A and WNT5A-induced migration in trans-well assays, with SK-N-AS only being susceptible to WNT5A and SK-N-DZ reacting also to WNT3A. Phosphorylation of the downstream PCP target RAC1 follows the same pattern. RAC1 is also induced by WNT3A in SK-N-DZ, indicating once more that WNT molecules cannot easily be attributed to a single WNT signaling pathway.

## 6. Are WNT Pathways Druggable in NB?

Even though the WNT signaling pathways offer attractive targets in many cancers, targeting of this pathway was shown to not only exert favorable effects (reviewed in [4,11]). In the case of adults, where development and proliferation are restricted to a limited number of tissue compartments, targets of WNT pathways, such as ROR1/2, which are rarely expressed in adult tissues, seem to be an attractive alternative or supplement to the conventional therapies (reviewed in: [102,103]). For NB, we have so far seen—that although WNT signaling is far from being well understood—some targets seem to emerge. Especially β-catenin and GSK-3β seem to be promising targets in MYCN-amplified NB because of the post-transcriptional down-regulation of MYCN protein levels, as outlined above. Also, tankyrase 1/2 inhibitors such as XAV935 and XAV939 may have promising effects in progressed and chemoresistant relapsed NB. They were reported to increase apoptosis by the induction of telomere shortening, prevent chemoresistance and inhibit WNT-β-catenin signaling, even in MYCN-amplified tumors [70,71,104,105]. However, NB is a tumor of children and most cases occur in young children. Therefore, targeting WNT signaling while many developmental processes that depend on this pathway are still ongoing, may not be recommended as a first line therapy. However, in cases of progressed chemoresistant *MYCN*-amplified tumors that are virtually untreatable with conventional regimens, targeting WNT signaling at the GSK-3β-β-catenin-tankyrase axis may be the last bullet. MYCN has been show to down-regulate WNT inhibitors such DKK1 and DKK3 [106,107]. For DKK3, the upregulation of miRNA-92 by MYCN has been shown to inhibit its expression. Targeting miRNA-92 may have promising effects on tumor proliferation and expansion, although targeting of MYCN with GSK-3β or tankyrase1/2 inhibitors should already include these effects.

## 7. Conclusions

WNT signaling in NB is far from being completely understood. The heterogenous results obtained in studies on NB cell lines reflect the general complexity of NB and its wide spectrum of clinical behavior. Defects in the WNT pathway might be causal for some of the lesions, while in others growth and metastasis formation may be WNT-dependent. Chromosomal aberrations such as deletions in chromosomes 1p or 11q are more frequently found in relapsed NB and in metastases. Of note, these chromosomes harbor WNT pathway members such as *WNT2B*, *WNT4*, *WNT11*, *ROR1*, *VANGL1* and others (reviewed by [22]). Loss of distinct WNT pathway components clearly has the potency to alter the behavior of the tumor cells and their environment. This underlines that WNT pathways offer promising targets for therapeutic interventions in NB. However, more data are required to elucidate the multitude of mutual interactions between WNT pathways and diverse other signaling cascades. Highly specific drugs are needed to prevent side effects of WNT targeting in growing and maturing tissues and organs of infants.

## Abbreviations

AKT	protein kinase B alpha
ALK	anaplastic lymphoma kinase
AP1	activator protein 1 /cJUN
APC	adenomatous polyposis coli protein
BAX	BCL2-associated X protein
BCL2	B-Cell CLL/Lymphoma 2
BDNF	brain-derived neurotrophic factor
BIM	BCL2 like 11
BMP	bone morphogenetic protein
CaMKII	calcium/calmodulin dependent protein kinase II
CD	cluster of differentiation
cMYC	v-Myc avian myelocytomatosis viral oncogene homolog
Cre	cyclization recombinase
CSK	casein kinase
DKK	dickkopf homolog
DVL	dishevelled
Eph	erythropoietin-producing hepatocellular carcinoma tyrosine kinase
ERK	mitogen-activated protein kinase P42
FZD	frizzled (G protein-coupled) receptor
GSK-3	glycogen synthase kinase-3
HGF	hepatocyte growth factor
JNK	c-Jun N-terminal kinase
Int	integration site 1 family member
LGR	leucine-rich repeat-containing G protein-coupled receptor
LRP	low density lipoprotein receptor-related protein
MAP-kinase	mitogen-activated protein kinase (MAPK)
MDR	multi drug resistance
MEK	MAPK/ERK activator kinase
mTOR	mechanistical (mammalian) target of rapamycin
MYCN	v-myc avian myelocytomatosis viral oncogene neuroblastoma-derived
NB	neuroblastoma
NC	neural crest
NCSC	neural crest stem cells
NF-κB	nuclear factor-κB
NFAT	nuclear factor of activated T-cells
NGF	nerve growth factor
Nrp	neuropilin
P53	tumor suppressor protein 53 (kD)
PCF11	pre-mRNA cleavage complex 2 protein
PCP	planar cell polarity
PI3K	phosphoinositide 3-kinase
PKC	protein kinase C
RAC	RAS-related C3 botulinum toxin substrate 1
RAF	rat fibrosarcoma serine/threonine kinase
RAS	rat sarcoma proto-oncogene, GTPase
RHOA	RAS homolog family member A
RNAi	RNA interference (by shRNA)
ROR	receptor tyrosine kinase-like orphan receptor
RSPO	R-spondin
RYK	related to receptor tyrosine kinase
Sema	semaphorin
sh	small hairpin (RNA)
SLC34A2	solute carrier family 34 member 2
TCF/LEF	t-cell factor / lymphoid enhancer factor (transcription factor)
TGF-β	transforming growth factor-β
TH	tyrosine hydroxylase
TNKS	tankyrase
TRIM59	tripartite motif containing 59
TRKA/B	neurotrophic receptor tyrosine kinase 1/2 (NTRK1/2)
VANGL	van Gogh like
WIF	WNT inhibitory factor
WNT	wingless-type MMTV integration site family
